# PATIENT-SPECIFIC DOSE ESTIMATES IN DYNAMIC COMPUTED TOMOGRAPHY MYOCARDIAL PERFUSION EXAMINATION

**DOI:** 10.1093/rpd/ncab016

**Published:** 2021-03-10

**Authors:** V-M Sundell, M Kortesniemi, T Siiskonen, A Kosunen, S Rosendahl, L Büermann

**Affiliations:** HUS Medical Imaging Center, Helsinki University Central Hospital, Helsinki, Uusimaa, Finland; Department of Physics, University of Helsinki, P.O. Box 64, 00014 University of Helsinki, Finland; HUS Medical Imaging Center, Helsinki University Central Hospital, Helsinki, Uusimaa, Finland; STUK-Radiation and Nuclear Safety Authority, Laippatie 4, Helsinki 00880, Finland; STUK-Radiation and Nuclear Safety Authority, Laippatie 4, Helsinki 00880, Finland; Department 6.2 Dosimetry for radiation therapy and diagnostic radiology, Physikalisch-Technische Bundesanstalt, Bundesallee 100, Braunschweig 38116, Germany; Department 6.2 Dosimetry for radiation therapy and diagnostic radiology, Physikalisch-Technische Bundesanstalt, Bundesallee 100, Braunschweig 38116, Germany

## Abstract

The study aimed to implement realistic source models of a computed tomography (CT) scanner and Monte Carlo simulations to actual patient data and to calculate patient-specific organ and effective dose estimates for patients undergoing dynamic CT myocardial perfusion examinations. Source models including bowtie filter, tube output and x-ray spectra were determined for a dual-source Siemens Somatom Definition Flash scanner. Twenty CT angiography patient datasets were merged with a scaled International Commission on Radiological Protection (ICRP) 110 voxel phantom. Dose simulations were conducted with ImpactMC software. Effective dose estimates varied from 5.0 to 14.6 mSv for the 80 kV spectrum and from 8.9 to 24.7 mSv for the 100 kV spectrum. Significant differences in organ doses and effective doses between patients emphasise the need to use actual patient data merged with matched anthropomorphic anatomy in the dose simulations to achieve a reasonable level of accuracy in the dose estimation procedure.

## INTRODUCTION

Accurate evaluation of patient-specific dose in computed tomography (CT) is a challenging task due to highly varying characteristics in patient anatomy and scan parameters. Rough order-of-magnitude estimates of effective dose are possible using dose-length product and body region-specific normalised effective dose conversion coefficients, acknowledging the limitations of the traditional computed tomography dose index (CTDI) formalism.^(1)^ Size-specific dose estimates (SSDEs) can be used to increase the patient-specific accuracy of the mean physical dose values within the scan range.^(1)^ However, these methods do not take into account patients’ unique properties and anatomy in an accurate way. In high-dose and high-risk applications, more accurate estimates of patient exposure are needed to e.g. avoid detriment in repeated procedures and as a basis for radiological optimization. Monte Carlo simulations are conducted with real patient data complemented with matched anthropomorphic phantom model data^(1)^ to enable accurate organ and effective dose estimates at a patient-specific level.

Many Monte Carlo software packages are available that can be used to simulate patient exposure to photons and electrons. However, CT scanner-specific information is needed for MC simulations. Not all needed information is readily available, but it is possible to measure the properties of missing parameters such as x-ray spectra and bowtie filter shape. Measured CT scanner radiation dose output is required to connect simulations to real patient dose. In this study, the CT scanner information is acquired with the procedure developed and tested in an earlier study,^(2)^ where it was demonstrated that accuracy of dose (simulation vs. measurement) to within 10% is achievable in the applied anthropomorphic phantoms.

There are several non-invasive techniques that allow the evaluation of myocardial perfusion. Myocardial perfusion can be studied with ultrasound (stress echocardiography), magnetic resonance imaging (stress CMR) and nuclear medicine imaging techniques like positron-emission tomography and single-photon emission CT.^(3–5)^ Recent advancements make myocardial perfusion imaging increasingly feasible also in CT.^(4–6)^ CT-myocardial perfusion imaging (CT-MPI) can be performed in two ways: static (snapshot, conventional) CT-MPI and dynamic CT-MPI.^(5,7)^ CT-MPI examination starts with intravenous iodinated contrast administration. In static mode, a single CT dataset of the left ventricular myocardium is acquired at the estimated time of myocardial enhancement peak. In dynamic mode, many CT datasets of the left ventricular myocardium are acquired. This enables the generation of time-attenuation curves.^(5)^

In earlier studies, mean effective dose values between 3.8 and 12.8 mSv, with an average of 9.23 mSv, have been reported for dynamic CT-MPI examinations.^(8)^ These values are based on the dose-length product and body region-specific normalised effective dose conversion coefficients. Variability is high due to the different CT scanners and imaging protocols used. For static examinations, significantly lower doses were reported, with an average of 5.93 mSv,^(8)^ but a large variation between 1.9 and 15.7 mSv existed.

The main purpose of this work was to implement earlier equivalent source model measurement methods and Monte Carlo simulations to real patient data merged with ICRP voxel phantom^(9)^ to examine the spread of patient-specific dose estimations under otherwise identical CT-MPI scan protocols with the same scanner.

## MATERIALS AND METHODS

Organ and effective doses were estimated for 20 patients in dynamic CT myocardial perfusion examination. To estimate organ doses outside the imaged region and effective doses more accurately, patient data were merged with ICRP 110 voxel phantom data.^(9)^ All data preprocessing, segmentation and data analysis were conducted with MATLAB (MathWorks, Natick, MA, USA).

### Patient and phantom data

Patient data comprise 20 CT angiography (CTA) axial image volumes. These 20 patients were women aged 40–84 years. Female patients were chosen in order to include highly radiation-sensitive breast tissue in the study context. Furthermore, breasts are directly irradiated organs in cardiac CT examinations. Consistent with the patient data, a reference ICRP voxel phantom model representing an adult female was used in simulations. Images were taken in HUS Medical Imaging Center with Somatom Definition Flash dual-source CT scanner (Siemens Healthcare, Forchheim, Germany). The pixel size of the data varied between patient scans, but the slice thickness was 3 mm in all cases. The pixel size of the ICRP phantom was 1.775 mm, and the slice thickness was 4.84 mm.^(9)^ The length of the imaged area varied, yielding 34–80 images per patient. In this study, we did not have information on patient weight or height, so we used the maximum effective diameter to get an estimation of patient size. The effective diameter was calculated as follows:

(1) \begin{equation*} \mathrm{effective}\ \mathrm{diameter}=\sqrt{\mathrm{LAT}\cdot \mathrm{AP}}, \end{equation*}

where $\mathrm{LAT}$ refers to the lateral dimension and $\mathrm{AP}$ refers to the anterior–posterior dimension of the patient.^(10)^ The effective diameter was calculated slice by slice, and the maximum value was used. The effective diameters of the patients, calculated after the missing tissue values were added, are listed in Table 1.

**Table 1:** Effective diameter of patients

Patient number	Effective diameter (mm)
1	313
2	331
3	320
4	272
5	276
6	292
7	245
8	256
9	297
10	251
11	322
12	316
13	342
14	312
15	313
16	329
17	332
18	304
19	322
20	354

### Data preprocessing and phantom matching

The CTA image stack consists only of a small part of the human body. The applied dosimetric patient model should include the whole body to take into account the scatter dose component outside of the scanned region. Therefore, the ICRP 110 female voxel phantom data was merged with the patient CTA data. For most of the images, the outer adipose tissue did not exist on images because of the small field of view. Missing adipose tissue was added to patients’ images if it was deemed necessary. Added adipose tissue had the shape of a circle segment in axial slice. However, one image stack contains only the inner part of the body, leaving most of the muscles, adipose tissue and breast out of the field of view. In this case, the outer part of the body was replaced by the ICRP phantom data.

**Figure 1:**
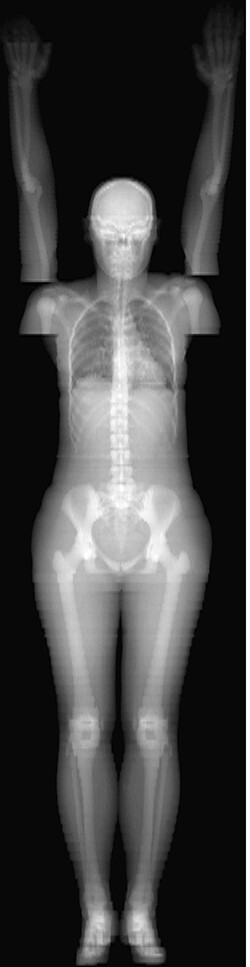
Projection image of the modified ICRP 110 voxel phantom

The ICRP 110 phantom data were transformed into phantom-based Hounsfield unit (HU) values. Values of −1000 HU for air, −700 HU for lungs, −100 HU for adipose tissue, 30 HU for soft tissue, 200 HU for blood and 1000 HU for bone were used. Cortical bone, spongiosa and medullary cavity were considered to be bone tissue. During the imaging, the patients were positioned arms up. Since in the ICRP phantom the arms and hands are next to the body, it was modified to an arms up position (Figure 1).

**Figure 2:**
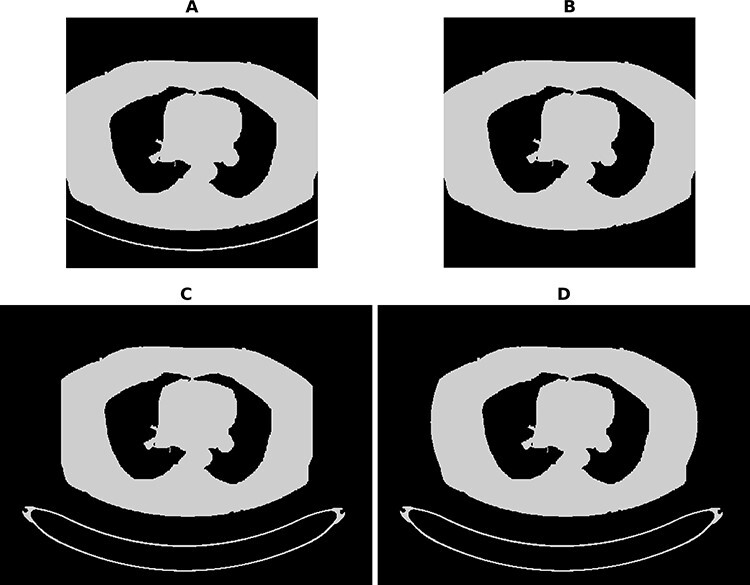
Schematic axial slice image of patient in different preprocessing steps: original image (**A**), image with removed partial patient support table, (**B**) image with added patient support table (**C**) and image with added adipose tissue (**D**)

In most CT images, the patient support table is partially visible, whereas the ICRP phantom does not include the patient table. The table was removed from the CT images as the first preprocessing step. In the second step, the patient and ICRP phantom data were resampled to have the same pixel size of 0.98 mm. During the resampling process, the slice thickness of the ICRP phantom was also resampled to 3 mm. In the third step, corresponding slices between patient data and ICRP phantom data were identified. In the fourth step, the ICRP phantom data were rescaled in such a way that cross-sectional areas of the bodies were the same at the lower junction point. In all resampling and rescaling processes, the nearest-neighbour interpolation method was used. In the fifth step, patient and ICRP phantom data were merged. In the sixth step, the patient table was added to the images. In the final step, the missing adipose tissue was added to the patient images if needed. Figure 2 shows schematic axial slice images of a patient in different preprocessing steps. Data preprocessing can take from a few minutes to an hour depending on the dataset. Most of the processes are automatically done, but finding the correct slices from the ICRP phantom to intersect with the patient and phantom data and adding adipose tissue still require manual help from the user.

### Segmentation

Patient organ segmentation was performed partially manually and partially based on HU values. For the ICRP phantom data, the original segmentation was used. In the manual segmentation of the CT image, the organ was delineated slice by slice. In the HU method, voxels in certain areas with predetermined value ranges were associated with that organ. The manual method and the HU-based method were used together for most of the datasets: target tissue/organ was first manually delimited roughly and then in that area the correct HU values for the particular tissue/organ were chosen. Lymphatic nodes could not be segmented from the patient data due to their small size. Therefore, only the ICRP phantom results were used to estimate absorbed dose for lymphatic nodes. This segmentation method is time-demanding due to the manual segmentation. Depending on the size of the image stack and the quality of segmentation required, it can take up to a few hours per patient.

### Source models

**Figure 3:**
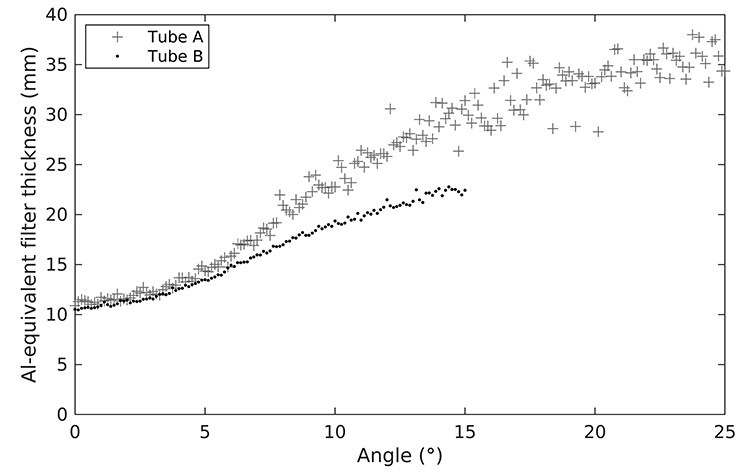
Al-equivalent bowtie filter thicknesses with inherent filtering for 80 kV spectrum for tube A and tube B for a Somatom Definition Flash

Source models for the Somatom Definition Flash were used in this study. Bowtie filter thicknesses for tube A and tube B were acquired on site. COBRA formalism (characterisation of bowtie relative attenuation) was used to determine bowtie filter shape, and x-ray spectra were calculated using aluminium attenuation measurements together with SpekCalc software tool and iterative algorithms.^(2,11,12)^ The bowtie attenuation characteristics with inherent filtering were measured for the 80 kV spectrum for tube A and tube B and Al-equivalent bowtie filters were calculated, and the resulting thickness is used in the simulation presented in Figure 3.

### Simulations

Monte Carlo simulations were performed with ImpactMC software (CT Imaging GmbH, Erlangen, Germany). ImpactMC is designed especially for Monte Carlo simulations of the 3D dose distribution in CT diagnostics. ImpactMC needs a CT image stack and scan parameters as input. Simulations were performed for the 20 patient data cases, including the scaled and matched ICRP phantom and the regular ICRP phantom. For ICRP phantom data simulation, the blood HU value was changed to 500 HU to simulate blood with iodinated contrast agent.

To simulate shuttle mode imaging, two simulations were made per image stack, one simulation for tube A and another simulation for tube B. The resulting dose maps from the two simulations were merged to get the total absorbed dose map. In dynamic study, several images are taken and starting angle can vary between image acquisitions; therefore, two full rotations were used in the simulations to average the starting angle variations. The beam collimation was 38.4 mm. Rotations were overlapping 10% such that the table increment was 34.56 mm (i.e. pitch = 0.9) and the total length of the imaged area was ~73 mm. The estimated absorbed dose was multiplied by the factor 95/360 since the shuttle mode uses an actual rotation angle of only 95°. Simulations were made for 80 and 100 kV spectra. Based on Siemens instructions, 370 mAs exposure was used for 80 kV simulations and 300 mAs exposure for 100 kV simulations. The distance from the focus to the centre of rotation was 595 mm, and the fan angle for tube A was 0.7955 rad and for tube B 0.5441 rad. Values for the Air KERMA free-in air at the iso-centre were measured^(2)^ and used for the simulation: 5.66 mGy/(100 mAs) was applied for 80 kV simulations and 10.35 mGy/(100 mAs) for 100 kV simulations. The bowtie filter model for the 80 kV spectrum was used in the simulations. Fifteen points on the time-attenuation curve were simulated, and thus, the simulated doses were multiplied by the factor 15.

In each simulation, $15\times{10}^9$ photons were simulated. Large numbers of photons were simulated to obtain better statistical accuracy in the low dose area. The values for the number of interactions and the cut-off energy were set to 10 and 10 keV, respectively. The number of projections per rotation was 36. Simulation parameters were chosen based on the ImpactMC manual to have good speed and accuracy for simulation. Simulations took less than a half-hour using GPU acceleration with NVIDIA GeForce GTX 1060 6 GB GPU. Size of the image stack affects the duration of the simulation. Different iodine concentrations on blood forced to make many inputs to material conversion tables. Material conversions are presented in Table 2. The maximum blood-iodine mixture HU value was determined as follows:

**Table 2:** Input definition for material conversion. Not all blood-iodine mixtures were needed for all simulations. For patient 4 (p4) blood-iodine mixtures 1…7 were needed, for patient 16 (p16) blood-iodine mixtures 1…12 were needed, for patient 18 (p18) blood-iodine mixtures 1…11 were needed and for the other patients blood-iodine mixtures 1…6 were needed. Possible observed range differences are presented as secondary range with the corresponding patient case number in parentheses

Material	Input (HU)
Air	−1024…−900
Lung	−899…−300
Adipose_tissue_(ICRU110Female)	−299…−50
Muscle_tissue_(ICRU110Female)	−49…199
Blood-iodine 1	200…249
Blood-iodine 2	250…299
Blood-iodine 3	300…349
Blood-iodine 4	350…399
Blood-iodine 5	400…449
Blood-iodine 6	450…499, 450…510 (p13)
Blood-iodine 7	500…549
Blood-iodine 8	550…599
Blood-iodine 9	600…649
Blood-iodine 10	650…699
Blood-iodine 11	700…749, 700…755 (p18)
Blood-iodine 12	750…771
Bone	500…1999, 510…1999 (p13), 536…1999 (p4), 756…1999 (p18), 772…1999 (p16)
Iron	2000…64535

(1) If >10% of the blood had HU values >501 HU, the maximum blood HU value was the value containing 90% of the blood voxels.

(2) If the maximum blood HU value was <501 HU, the maximum blood HU value was the maximum value.

(3) Otherwise, the maximum blood HU value was 499 HU.

Iodine concentration in the blood was calculated based on the HU value. The HU value of blood without iodine was assumed to be 40 HU. The relationship between the iodine concentration and the contrast enhancement was taken from the previous study by Bae *et al*.^(13)^ For 80 kV, 41.12 HU/(mg iodine/ml) and for 100 kV 31.74 HU/(mg iodine/ml) were used. Table 3 shows the mass percentage of blood and iodine in all blood-iodine mixtures.

**Table 3:** Mass percentage of blood and iodine in all blood-iodine mixtures used for 80 and 100 kV simulations

Material	Blood (%)		Iodine (%)	
	80 kV	100 kV	80 kV	100 kV
Blood-iodine 1	99.558	99.419	0.442	0.581
Blood-iodine 2	99.430	99.261	0.570	0.739
Blood-iodine 3	99.308	99.104	0.692	0.896
Blood-iodine 4	99.187	98.946	0.813	1.054
Blood-iodine 5	99.065	98.789	0.935	1.211
Blood-iodine 6	98.943	98.631	1.057	1.369
Blood-iodine 7	98.822	98.474	1.178	1.526
Blood-iodine 8	98.700	98.316	1.300	1.684
Blood-iodine 9	98.579	98.158	1.421	1.842
Blood-iodine 10	98.457	97.940	1.543	2.060
Blood-iodine 11	98.336	97.843	1.664	2.157
Blood-iodine 12	98.248	97.604	1.752	2.396

### Absorbed and effective doses

Absorbed doses for different tissues were calculated from simulations as a mean dose of segmented voxels. However, the absorbed dose of red bone marrow (rbm) was estimated in the patient data area using bone segmentation and in the ICRP phantom area using the spongiosa segmentation that contains rbm. The amount of spongiosa in the patient data area was estimated to be the same as in the ICRP phantom, so the number of segmented bone voxels was multiplied by a correction coefficient when calculating the absorbed dose for rbm. The mass-energy absorption coefficient method (MEAC method) was used to transform the bone dose in patient data to the rbm dose. The estimated absorbed dose for rbm was calculated as follows:

(2) \begin{equation*} {D}_{\mathrm{rbm}}=\frac{{\left(\frac{\mu_{\mathrm{en}}}{\rho}\right)}_{\mathrm{rbm}}}{{\left(\frac{\mu_{\mathrm{en}}}{\rho}\right)}_{\mathrm{bone}}}{D}_{\mathrm{bone}}, \end{equation*}

where ${D}_{\mathrm{rbm}}$ is the absorbed dose for the rbm and ${D}_{\mathrm{bone}}$ is the absorbed dose for the bone, including all compositional structures, and $\frac{\mu_{\mathrm{en}}}{\rho }$ the MEAC.^(14)^ MEACs were calculated using the equation below:

(3) \begin{equation*} {\left(\frac{\mu_{en}}{\rho}\right)}_x=\sum_{E={E}_{\mathrm{min}}}^{E_{\mathrm{max}}}\sum_{m={m}_1}^{m_y}\frac{\mu_{en}}{\rho}\left(m,E\right)\cdot{R}_m(m)\cdot{R}_E(E), \end{equation*}

where *E* refers to photon energy, *m* refers to elemental materials, *R_m_* is the relative portion of elemental material on tissue *x* and *R_E_* is the relative portion of energy on spectrum. MEACs were interpolated from data with a 1 keV interpolation interval using cubic spline interpolation. Ratios of mean MEACs were calculated for the incident 80 and 100 kV spectra (i.e. before photons reach the patient) using rbm and bone elemental composition and MEACs acquired from the NIST database.^(15)^ The rbm-to-bone ratio of the mean MEACs for the 80 kV spectrum was 0.2761 and for the 100 kV spectrum 0.3014.

The effective dose was calculated as follows:

(4) \begin{equation*} E=\sum_i{w}_i\cdot{D}_i, \end{equation*}

where ${w}_i$ is the weighting factor for tissue or organ *i* based on the ICRP 103 publication and ${D}_i$ the corresponding mean absorbed dose^(16)^.

## RESULTS

**Figure 4:**
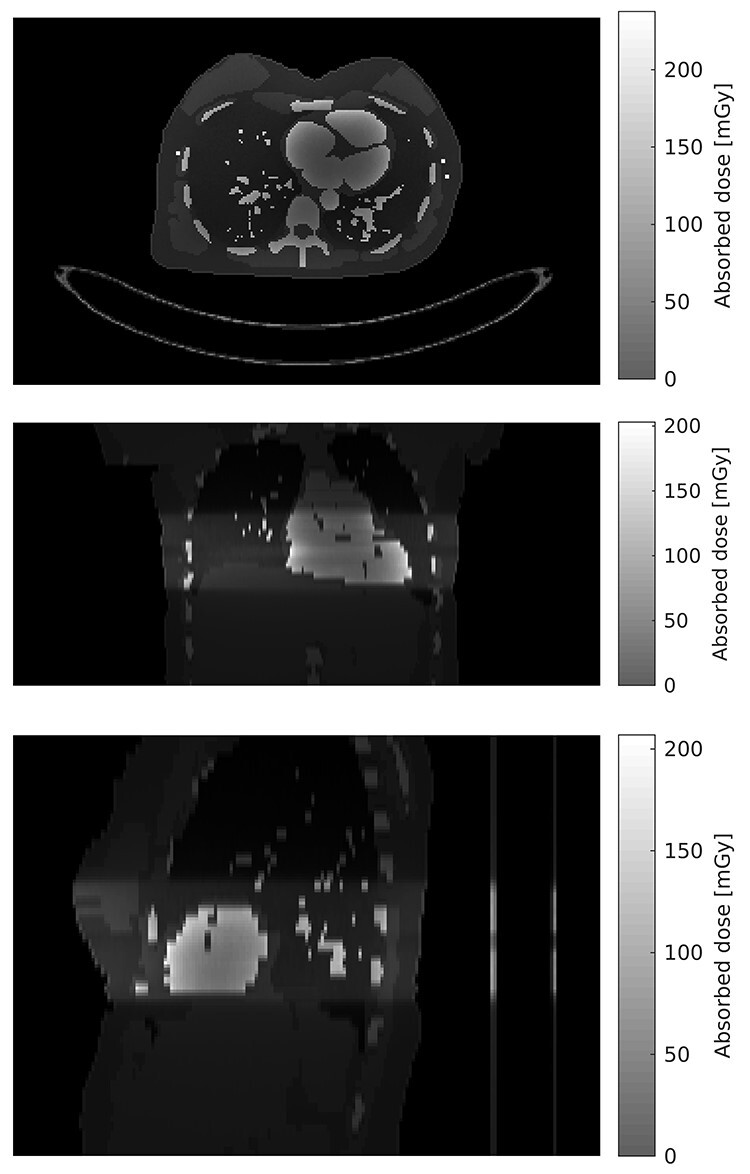
Simulated absorbed dose distribution with 80 kV spectrum for modified ICRP phantom

Patient-specific absorbed doses and effective doses are presented in Tables 4 and 5 for 80 and 100 kV simulations, respectively. Figure 4 shows simulated absorbed dose distribution with the 80 kV spectrum for modified ICRP phantom. Patients can be separated into two groups: those whose effective diameter is less than or equal to 32 cm and those whose effective diameter is >32 cm. The grouping is based on the Siemens instructions recommending that 80 kV tube voltage with 370 mAs exposure is suitable for patients with a thorax diameter of no >32 cm. Based on the same instructions, 100 kV tube voltage with 300 mAs is suitable for patients with a thorax diameter of >32 cm. Selected patients for the 80 kV group are therefore patient numbers 1, 3, 4, 5, 6, 7, 8, 9, 10, 12, 14, 15 and 18. The remainder comprise patients for the 100 kV group. Table 6 shows the mean effective dose and mean absorbed dose ± standard deviation for tissues/organs with mean absorbed dose >5 mGy in 100 kV simulations. Results are calculated based on all 80 and 100 kV simulations and also based on selected patients for the 80 and 100 kV groups.

**Table 4:** Patient-specific absorbed doses and effective doses for 80 kV simulations

	Absorbed dose (mGy)																											
Patient number	Adrenals	Bones	Brain	Breasts	Colon	Nasal passage extrathoratic (ET)	Gall bladder	Gonads	Heart	Kidneys	Liver	Lungs	Lymphatic nodes	Muscle	Oesophagus	Oral mucosa	Pancreas	rbm	Salivary glands	Skin	Small intestine	Spleen	Stomach	Thymus	Thyroid	Urinary bladder	Uterus	Effective dose (mSv)
1	2.91	5.39	0.05	13.27	0.10	0.43	2.62	0.01	27.36	1.33	7.22	22.99	3.88	1.20	7.89	0.56	1.40	2.94	0.50	1.17	0.38	6.08	4.98	4.57	1.77	0.00	0.01	6.25
2	2.49	5.14	0.05	15.62	0.09	0.43	2.25	0.01	26.28	1.14	7.63	20.64	3.88	0.94	13.76	0.55	1.22	2.78	0.48	1.11	0.34	6.64	3.58	3.94	1.49	0.00	0.01	6.27
3	2.20	5.55	0.03	16.03	0.07	0.28	1.92	0.01	27.10	1.00	4.84	22.95	3.88	1.29	9.68	0.36	1.03	2.92	0.31	1.00	0.28	4.48	2.88	3.29	1.17	0.00	0.01	6.27
4	2.58	6.68	0.03	22.22	0.09	0.29	2.29	0.01	37.80	1.13	7.10	25.48	3.88	1.66	13.26	0.36	1.20	3.68	0.33	1.54	0.33	6.86	4.30	2.15	1.27	0.00	0.01	7.92
5	8.56	5.68	0.03	16.71	0.19	0.29	3.53	0.01	46.59	2.67	10.51	26.18	3.88	1.89	15.73	0.35	2.44	3.38	0.32	1.41	0.52	7.80	5.35	2.57	1.43	0.01	0.01	7.73
6	2.93	5.60	0.03	25.18	0.10	0.29	2.54	0.01	38.23	1.27	8.25	25.31	3.88	1.45	12.99	0.37	1.32	3.10	0.30	1.25	0.36	10.03	5.27	2.28	1.29	0.00	0.01	8.27
7	2.51	6.98	0.04	67.20	0.08	0.35	2.09	0.01	37.47	1.09	7.09	34.14	3.88	1.93	15.37	0.45	1.10	3.76	0.39	2.06	0.30	6.08	5.21	3.84	1.45	0.00	0.01	14.56
8	2.99	7.18	0.04	22.15	0.09	0.30	2.60	0.01	39.42	1.27	8.20	31.97	3.88	1.83	16.31	0.39	1.35	3.87	0.34	1.50	0.36	6.19	4.45	3.16	1.24	0.00	0.01	8.91
9	2.34	6.14	0.03	14.80	0.08	0.31	2.06	0.01	32.44	1.04	5.98	24.54	3.88	1.33	13.39	0.40	1.09	3.28	0.33	1.12	0.29	4.61	3.04	3.76	1.28	0.00	0.01	6.60
10	3.84	7.65	0.04	13.25	0.12	0.35	3.42	0.01	46.49	1.61	11.77	33.54	3.88	2.76	17.77	0.45	1.75	4.26	0.40	1.71	0.46	10.32	7.38	3.14	1.36	0.01	0.01	8.75
11	2.62	5.17	0.05	27.60	0.10	0.44	2.38	0.01	25.48	1.21	6.39	22.18	3.88	1.69	10.08	0.58	1.28	2.75	0.51	1.21	0.35	5.36	3.60	4.37	1.60	0.00	0.01	7.77
12	2.52	5.56	0.05	27.27	0.09	0.41	2.23	0.01	31.16	1.16	6.50	22.86	3.88	1.48	9.83	0.53	1.24	3.03	0.45	1.15	0.34	5.56	4.31	4.13	1.47	0.00	0.01	7.93
13	3.12	4.21	0.04	36.98	0.15	0.36	2.86	0.01	18.26	1.55	7.62	15.80	3.88	0.85	7.19	0.47	1.65	2.35	0.38	0.99	0.47	6.96	3.46	2.63	1.08	0.01	0.01	7.88
14	4.24	5.14	0.03	27.75	0.17	0.26	3.89	0.01	31.00	1.92	9.74	20.63	3.88	1.41	12.69	0.34	2.08	2.85	0.29	1.31	0.57	12.02	5.21	2.63	1.03	0.01	0.01	8.03
15	2.73	5.61	0.04	15.39	0.11	0.36	2.53	0.01	31.58	1.24	6.78	24.22	3.88	1.41	12.42	0.46	1.37	2.98	0.41	1.25	0.37	5.46	3.71	2.82	1.29	0.01	0.01	6.71
16	2.83	4.90	0.04	10.49	0.12	0.34	2.62	0.01	23.60	1.37	7.53	17.29	3.88	1.15	6.58	0.45	1.48	2.69	0.38	0.99	0.41	6.62	3.55	2.45	1.07	0.01	0.01	4.95
17	2.30	4.95	0.07	18.54	0.09	0.54	2.07	0.01	29.42	1.08	6.43	21.35	3.88	1.23	9.15	0.70	1.12	2.66	0.62	1.07	0.31	5.35	4.64	5.07	1.88	0.00	0.01	6.61
18	3.45	6.66	0.04	27.70	0.13	0.33	3.06	0.01	32.07	1.55	8.19	24.88	3.88	1.21	9.43	0.43	1.66	3.66	0.38	1.21	0.45	6.84	5.56	2.16	1.14	0.01	0.01	8.46
19	2.62	5.72	0.03	10.13	0.09	0.31	2.34	0.01	36.18	1.17	6.83	23.27	3.88	1.58	11.82	0.40	1.24	3.06	0.36	1.12	0.34	5.05	3.41	3.23	1.22	0.00	0.01	5.91
20	2.84	3.92	0.04	5.12	0.12	0.39	2.58	0.01	23.14	1.35	6.57	18.61	3.88	0.92	9.22	0.49	1.46	2.19	0.41	0.88	0.41	6.03	3.46	2.89	1.60	0.01	0.01	4.44
ICRP	3.61	5.35	0.03	46.81	0.13	0.27	3.38	0.01	30.28	1.61	11.59	25.79	3.88	1.97	11.13	0.35	1.69	3.19	0.29	3.00	0.45	7.62	5.89	2.94	0.97	0.00	0.00	11.13

**Table 5:** Patient-specific absorbed doses and effective doses for 100 kV simulations

	Absorbed dose (mGy)																											
Patient number	Adrenals	Bones	Brain	Breasts	Colon	Nasal passage extrathoratic (ET)	Gall bladder	Gonads	Heart	Kidneys	Liver	Lungs	Lymphatic nodes	Muscle	Oesophagus	Oral mucosa	Pancreas	rbm	Salivary glands	Skin	Small intestine	Spleen	Stomach	Thymus	Thyroid	Urinary bladder	Uterus	Effective dose (mSv)
1	5.83	10.08	0.13	22.82	0.25	0.89	5.25	0.02	47.90	2.72	13.18	39.94	6.91	2.15	14.29	1.12	2.87	6.00	1.00	2.06	0.80	11.23	9.18	8.42	3.47	0.01	0.02	11.09
2	5.00	9.77	0.13	26.77	0.22	0.86	4.54	0.02	46.43	2.36	13.95	36.10	6.91	1.70	24.70	1.10	2.52	5.78	0.98	1.93	0.71	12.27	6.80	7.36	2.95	0.01	0.02	11.16
3	4.35	10.32	0.08	27.52	0.18	0.58	3.82	0.02	47.22	2.06	8.88	39.53	6.91	2.29	16.93	0.73	2.12	5.94	0.63	1.79	0.60	8.29	5.41	6.14	2.31	0.01	0.01	11.03
4	5.07	11.91	0.09	37.98	0.21	0.59	4.49	0.02	64.79	2.28	12.75	43.48	6.91	2.88	23.27	0.73	2.44	7.17	0.66	2.66	0.69	12.55	7.92	3.99	2.42	0.01	0.02	13.79
5	16.20	9.32	0.08	28.61	0.42	0.59	6.79	0.03	79.50	5.24	18.67	44.84	6.91	3.31	27.38	0.70	4.76	6.04	0.65	2.44	1.05	14.10	9.84	4.68	2.73	0.02	0.03	13.43
6	5.78	9.79	0.08	43.29	0.23	0.59	5.03	0.02	65.80	2.58	14.94	43.62	6.91	2.56	22.69	0.75	2.70	5.91	0.62	2.19	0.74	18.60	9.61	4.26	2.49	0.01	0.02	14.44
7	4.87	11.37	0.11	112.76	0.18	0.70	4.07	0.02	63.76	2.17	12.95	57.65	6.91	3.36	26.21	0.88	2.22	6.67	0.76	3.50	0.62	10.92	9.29	6.97	2.77	0.01	0.02	24.68
8	5.75	13.16	0.09	37.62	0.22	0.61	5.07	0.02	67.24	2.53	14.57	53.74	6.91	3.16	27.71	0.78	2.70	7.75	0.69	2.62	0.74	11.06	8.03	5.81	2.37	0.01	0.02	15.33
9	4.64	11.07	0.09	25.56	0.19	0.65	4.13	0.02	56.24	2.14	10.93	42.23	6.91	2.35	23.44	0.82	2.25	6.46	0.69	1.99	0.62	8.50	5.69	6.93	2.52	0.01	0.02	11.60
10	7.34	12.95	0.10	22.59	0.28	0.69	6.57	0.02	78.83	3.16	20.70	56.36	6.91	4.73	30.29	0.90	3.45	7.87	0.78	2.94	0.93	18.06	13.11	5.77	2.61	0.01	0.02	15.00
11	5.26	9.55	0.13	47.18	0.23	0.90	4.79	0.02	45.08	2.50	11.74	38.79	6.91	3.02	18.09	1.15	2.64	5.56	1.02	2.11	0.74	9.97	6.78	8.19	3.20	0.01	0.02	13.69
12	5.03	10.27	0.12	46.70	0.22	0.83	4.49	0.02	54.67	2.38	12.00	39.83	6.91	2.64	17.60	1.07	2.56	6.11	0.90	2.03	0.72	10.30	8.05	7.70	2.91	0.01	0.02	13.96
13	6.24	8.11	0.11	63.02	0.34	0.72	5.75	0.02	32.86	3.18	14.17	27.99	6.91	1.56	13.27	0.96	3.39	4.95	0.79	1.75	0.98	12.87	6.63	5.13	2.21	0.02	0.02	13.90
14	8.29	9.62	0.08	47.50	0.37	0.56	7.58	0.03	54.15	3.88	17.67	35.98	6.91	2.52	22.56	0.70	4.19	5.82	0.60	2.30	1.17	21.65	9.66	5.00	2.08	0.02	0.03	14.16
15	5.41	10.46	0.10	26.54	0.26	0.74	5.04	0.02	55.27	2.53	12.43	41.72	6.91	2.50	21.70	0.95	2.81	6.06	0.83	2.20	0.78	10.00	6.93	5.34	2.52	0.01	0.02	11.81
16	5.69	9.68	0.10	18.09	0.27	0.70	5.22	0.02	41.77	2.81	13.85	30.33	6.91	2.07	12.12	0.91	3.04	5.81	0.78	1.76	0.86	12.18	6.73	4.75	2.18	0.01	0.02	8.92
17	4.68	9.51	0.16	31.98	0.22	1.11	4.20	0.02	51.96	2.25	11.81	37.55	6.91	2.23	16.73	1.41	2.36	5.60	1.23	1.89	0.67	10.05	8.67	9.49	3.74	0.01	0.02	11.82
18	6.71	12.57	0.10	47.19	0.29	0.67	6.03	0.02	55.71	3.11	14.84	42.67	6.91	2.14	16.85	0.86	3.35	7.55	0.77	2.13	0.93	12.31	10.07	4.13	2.25	0.02	0.02	14.79
19	5.16	10.22	0.09	17.54	0.21	0.64	4.69	0.02	62.42	2.38	12.41	40.07	6.91	2.80	20.48	0.82	2.55	5.97	0.73	1.98	0.70	9.28	6.33	6.00	2.42	0.01	0.02	10.40
20	5.68	7.59	0.11	9.21	0.27	0.80	5.24	0.02	41.48	2.79	12.23	32.48	6.91	1.66	16.52	1.00	3.03	4.64	0.85	1.55	0.86	11.10	6.60	5.45	3.15	0.01	0.02	8.04
ICRP	7.05	8.97	0.08	78.47	0.28	0.54	6.53	0.01	51.99	3.20	20.62	44.49	6.91	3.46	20.10	0.70	3.37	5.82	0.58	5.06	0.91	13.76	10.65	5.48	1.92	0.01	0.01	19.11

The highest mean absorbed doses were found for the heart and then for the lungs and breasts. However, the breasts have the highest standard deviation and the variability in breast doses is much higher than in doses in other tissues/organs. When comparing mean effective doses between 80 and 100 kV simulations in the case where all patients are included, the effective dose is 75% higher in 100 kV examinations. However, if the groups with the selected patients are compared, the effective dose is only 36% higher in 100 kV examinations. The combined mean effective dose for selected 80 and 100 kV examinations is 9.2 ± 2.5 mSv.

## DISCUSSION

Patient-specific dose estimate method can be used to evaluate accurately patient dose in CT. In this method, real patient data complemented with the matched anthropomorphic phantom model is used as input volume in Monte Carlo dose simulations. Taking into account patients’ unique anatomical properties, this method provides more accurate patient dose estimates at the individual level than SSDEs or dose-length product and effective dose conversion coefficients. For Monte Carlo simulations, bowtie filter shape, x-ray spectra and radiation dose output are needed. Methods to acquire these parameters have been developed and tested in an earlier study.^(2)^ In this study, Monte Carlo simulations and previously developed methods to acquire necessary scan parameters were used to evaluate patient-specific dose estimates for 20 patients in dynamic CT myocardial perfusion examination.

Li *et al*.^(18–21)^ have earlier evaluated patient-specific dosimetry and applied it to paediatric patients. However, no earlier studies exist on patient-specific dosimetry in myocardial perfusion examination. Dose estimations for myocardial perfusion examinations in various studies are based on dose-length product and effective dose conversion coefficients. Such conversion coefficients are only order-of-magnitude level estimates provided for rough anatomical regions such as the coefficients given in the EU RP 154 document.^(22)^ Danad *et al*.^(8)^ collected information about radiation dose in CT myocardial perfusion examinations. For dynamic studies, the effective dose varied from 3.8 to 12.8 mSv, with a mean value of 9.23 mSv. Fujita *et al*.^(23)^ investigated dose reduction in dynamic CT stress myocardial perfusion imaging and compared 80 kV with 370 mAs and 100 kV with 300 mAs protocols. Estimated effective dose for the 80 kV group was 6.1 ± 1.1 mSv and for the 100 kV group 10.7 ± 1.9 mSv. Compared with our results, these effective dose estimates are lower. However, patient characteristics were different from our study, and most importantly, the average number of acquisition phases was lower in their study, which has a proportional effect on patient dose.

The breasts and lungs have the largest contribution to the effective dose. However, marked variability in breast dose exists between patients. If the breast tissue is mostly in the scanned area, the absorbed dose will be high. This can be seen in the ICRP phantom data simulation where a large amount of breast tissue is in the scanned area. However, if only a small part of the breast tissue is located within the scan area, the mean breast dose will be substantially lower. This demonstrates the consequence of variable patient anatomy on the actual radiation doses to the patient, demonstrating the need for patient-specific dose estimates also in cardiac CT perfusion imaging.

When calculating the effective dose, correct organ segmentation is very important. In most clinical CT scans, as appropriate according to clinical indication and general optimization principles, the acquired CT scan data of the patient should cover only a part of the body. When applied to chest CT data, as in this study, or especially when applied to perfusion scan data of the heart, the missing part of the patient anatomy has to be approximated with an anthropomorphic model. The model should match the actual patient anatomy as closely as possible. In this study, the female patients were merged with the female ICRP phantom model to supplement the missing individual anatomy outside the patient chest CT scan data. Organ size, shape, orientation and location in the body are important parameters when estimating the absorbed radiation dose to organs. Individual sizes of organs vary significantly^(24,25)^ so an anthropomorphic phantom model, even a matched one, provides only a rough estimate of the real patient anatomy. Fortunately, the primary patient scan data represent the directly exposed anatomical area (excluding the helical overranging, which can be minimised by using dynamic helical collimation), and the supplementary phantom data are mainly used to determine the scatter contribution. This makes effective dose estimate more accurate than a situation where there is only phantom data.

**Table 6:** Mean effective dose and mean absorbed dose ± standard deviation for tissues/organs with mean absorbed dose >5 mGy in 100 kV simulations except lymphatic nodes

Tissue/organ	Absorbed dose (mGy)			
	80 kV all	80 kV (diameter ≤ 32 cm)	100 kV all	100 kV (diameter > 32 cm)
Adrenals	3.1 ± 1.4	3.4 ± 1.7	6.1 ± 2.6	5.4 ± 0.5
Bones	5.7 ± 1.0	6.1 ± 0.8	10.4 ± 1.5	9.2 ± 1.0
Breasts	21.7 ± 13.2	23.8 ± 14.2	37.0 ± 22.1	30.5 ± 18.8
Gall bladder	2.6 ± 0.5	2.7 ± 0.6	5.1 ± 1.0	4.9 ± 0.5
Heart	32.1 ± 7.5	35.3 ± 6.4	55.7 ± 12.1	46.0 ± 9.3
Liver	7.6 ± 1.6	7.9 ± 1.9	13.7 ± 2.7	12.9 ± 1.1
Lungs	23.9 ± 4.8	26.1 ± 4.3	41.2 ± 7.7	34.8 ± 4.6
Oesophagus	11.7 ± 3.2	12.8 ± 3.0	20.6 ± 5.1	17.4 ± 4.3
rbm	3.1 ± 0.5	3.4 ± 0.4	6.2 ± 0.9	5.5 ± 0.5
Spleen	6.7 ± 2.0	7.1 ± 2.3	12.3 ± 3.5	11.1 ± 1.4
Stomach	4.4 ± 1.1	4.7 ± 1.2	8.1 ± 1.9	6.9 ± 0.8
Thymus	3.3 ± 0.9	3.1 ± 0.8	6.1 ± 1.6	6.6 ± 1.8
Effective dose (mSv)	7.5 ± 2.1	8.2 ± 2.1	13.1 ± 3.4	11.1 ± 2.2

Manual segmentation is time-consuming. If personalised dosimetry is intended for general use for all patients after CT examinations, segmentations need to be done automatically. Deep learning and convolutional neural network might be useful for this purpose.^(26,27)^ As stated by Dong *et al*.,^(27)^ automatic segmentation with a deep-learning method can be performed for multiple organs accurately within a few seconds, whereas manual segmentation usually requires 30 minutes or more.

In this study, source models and patient data from Somatom Definition Flash were used, which limits effective dose estimates to that device alone. However, the method is generally applicable to any CT scanner and any scanned region, not limited to myocardial perfusion imaging.

### Uncertainties

There are several sources of uncertainty in the simulated doses. Sources of uncertainties are related to the simulation software, equivalent source models, input to material conversion, patient data and voxel phantom data. In simple phantom simulations with ImpactMC, when the Al-equivalent bowtie filter and spectra are determined and applied, as in this study, the maximum difference between measurement and simulation results has been ~15% and the typical difference some 5%. This level of accuracy is achievable for simple cases with a medium-sized thorax CT phantom (CIRS 007TE-17). Simulations with patient data are more complex, which can lead to higher uncertainties. Each individual set of patient data contains a large number of different tissues. Due to the basic elemental material properties and physical interactions, it is anticipated and also observed that the CT-number (HU) contrast scale of the tissues can overlap significantly. This is also reflected in the application of the material conversion table in the simulation. For studies where iodine contrast agent is used, the overlap of iodine contrast-enhanced blood and bones with calcium presents an overlap in the CT-number (HU) range. Therefore, some bone tissue with a low HU value was considered to be enhanced blood, and also some high HU value blood was considered to be bone tissue.

Patient CT image data may also incorporate different types of artefacts. Artefacts change HU values in an undesirable way, which may lead to errors in material conversion. If the artefacts are significant, HU values might need further correction in order to reach accurate results in dose simulations. Fortunately, the patient data in this study had only small metal artefacts, which had a minor effect on the results.

Obviously, the segmentation of patient data needs to be done accurately, particularly for organs that are only partially in the primary x-ray beam. Unfortunately, in this study, lymphatic nodes could not be segmented from the patient data. That is why the ICRP phantom simulations were used to estimate absorbed dose of lymphatic nodes. However, lymphatic nodes are distributed over the body, with only a small mass fraction of nodes in the imaged region. Therefore, lymphatic nodes have only a minor effect on the effective dose. Furthermore, rbm could not be segmented from CT images. Therefore, the MEAC method was used to estimate the absorbed dose.

The scaling method used to scale the ICRP phantom to the same size as the patient was simple. The method was selected because it was easy to implement. However, it has some shortcomings that should be taken into consideration when viewing the absorbed dose results for organs and tissues. The method scales all organs with the same scaling factor depending on the patient’s size relative to the ICRP phantom size. Thus, it fails to take into account the patient’s body composition. For a large patient, it is more probable that organs are scaled too much relative to the organs’ actual size. For patients with a size nearer to that of the ICRP phantom, the scaling method most likely works better than for larger patients. Also, organ locations on the ICRP phantom are fixed, and these locations might not correspond to the actual locations on a patient’s body.

The nominal x-ray beam collimation width was applied in the simulations in this study. However, the actual total collimation is slightly wider than the nominal collimation width. This can lead to an underestimation in dose estimates, especially for organs that are partly in the x-ray beam. Lack of the 100 kV Al-equivalent bowtie filter shape forced us to use the 80 kV Al-equivalent bowtie filter also for 100 kV simulations. However, based on our measurements and a study by Yang *et al*.,^(17)^ there should be only small differences between the 80 and 100 kV Al-equivalent bowtie filter shapes. Usage of the 80 kV Al-equivalent bowtie filter shape in all simulations should therefore be an adequate solution.

## CONCLUSION

Monte Carlo simulations with a realistic CT scanner model and phantom data-enhanced patient data were used to provide absorbed dose maps. Patient-specific organ doses and effective doses were determined from these dose maps. Significant differences in organ doses and effective doses between patients were found and can be explained by differences in patient data. This emphasises the need to use actual patient data merged with matched anthropomorphic anatomy in the dose simulations to achieve a reasonable level of accuracy in the dose estimation procedure.
